# Event and Comment

**Published:** 1922-12

**Authors:** 


### DENTAL REGISTER

THE MAGAZINE OF DENTISTRY

Vol. LXXVI DECEMBER, 1922 No. 12

### EVENT AND COMMENT

**Meeting of the Dental Society Ohio State** The annual meeting of the Ohio State Dental Society, which convened December five, six, and seven, 1922, in Cincinnati, was an unusual event, and its program was remarkably satisfactory for all who engaged in it, or witnessed the skillful conduct of its management. . The meeting was held in Cincinnati to make it possible to have the Callahan Memorial unveiled at that time. But the skillful conduct of the sessions, in connection with the meeting of the American Academy of Periodontology, at the same time, added much to the occasion; and demonstrated that Cincinnati dentists know how to entertain a dental convention, as few possess this art, to the entire satisfaction of those attending fully demonstrated.

The sessions of the Academy of Periodontology, being a national body, necessarily attracted interest from a wider distance, while the State Society attracted many by the varied program offered. Both societies co-operated so harmoniously that the entire convention was a constant and continual intellectual feast. It is remarkable that one could follow so closely an attention meeting without becoming either overfed, or fail to get a real and stimulating inspiration. In this instance it was the general verdict that this was one of the most inspiring dental meetings ever held in this Society. It is practically impossible for us to report this meeting without more detail than is practicable here. The character of the papers presented, and the discussions, and details of the clinics presented, were of much value and intensely interesting. The program committee and the officers who planned and carried on the meeting so admirably deserve great credit for the thoroughness with which they met the exacting task and so completely satisfied everybody.

The program as announced was carried out as advertised in the journals and no important feature was omitted. It would be impracticable to rehearse the program in detail—it would also be difficult even to specify the more conspicuous items for special mention. The program was so well ordered and balanced that it claimed the interest and attention of the entire convention because it had a real interest for everyone.

One of the unique and most interesting features was presented by the Oral Hygiene Section, which was given at the noon luncheon, with special emphasis on foods and diets. For lack of time this was not as thoughtfully presented as this subject deserved, but at any rate the subject was presented so well that it created a real interest in this work.

The feature of the meeting was the oral hygiene luncheon served at noon. A balanced diet, as prescribed by nationally known dietitians, was served.

Dr. Sidney J. Rauh presided at the luncheon and introduced a number of speakers, each limited to a five-minute speech. Among them were H. H. Snively, Columbus, health commissioner of Ohio, who discussed oral hygiene in the health program of Ohio; Ann L. Buntin, Cincinnati, who explained the work of social workers in urging better care of the teeth; Melville Quinby, Forsythe Institute, Mass., who spoke of dental and oral hygiene.

This is the menu of the ''oral hygiene luncheon'' arranged for the delegates to the convention of the Ohio State Dental Association, and which was served Tuesday at the Hotel Gibson: Green celery, boiled spinach, beans, brussels sprouts, carrots, cauliflower and cabbage, poached egg, whole wheat bread, butter, milk and fruit salad.

Sugar, condiment and vinegar was taboo. It was described as a "balanced diet."

The Callahan Memorial Monument was presented by appropriate exercises at the Cincinnati Hospital, where the beautiful monument is erected on the grounds of the hospital. The base is of marble, on which is mounted a bronze head that is a remarkable likeness of Dr. Callahan. It is the gift of Ohio dentists and his friends, who thus honor him for his devotion to the science for which he gave his time and means, if not actually his very life.

Another delightful incident was the presentation to Dr. J. Leon Williams of a gold medal, "The Callahan Award"—a token of regard for the scientific research which has characterized Dr. Williams' best energies, and given the profession much of its most valuable attainments at the present time. Surely a deserved tribute to an active worker in many fields.

---

### DENTAL HYGIENE

Before I lay me down to sleep,
Each night I'll brush my teeth,
I'll brush the front, I'll brush the back,
I'll brush on top and underneath.

Each morning when I awake,
Again my little brush I'll take,
A thorough brushing to repeat,
To keep my mouth clean and sweet.

At least twice, in each year,
Before my dentist I'll appear,
Resolved to follow his advice,
And keep my teeth polished nice.

—George N. West, D.D.S.

**Dentistry and the Unfortunate Poor**

Dentistry is a branch of medicine. We have copied the medical code of ethics, and in general try to conduct ourselves like our medical brothers; yet in one phase we differ from them very much, and this difference has often been pointed out by many physicians with considerable criticism.

The average medical man does a great deal of charity work, both in his office and in public institutions. Dispensaries, clinics, infirmaries, or hospitals could not exist if it were not for the generosity of the medical profession, but the dentists are seldom giving any of their time for nothing. A poor child if in need of an operation; say removal of tonsils or adenoids, can nearly always receive the necessary care, regardless of its parents' ability to pay, but the same child would find it rather difficult to get its teeth filled free of charge, unless it lived in Boston, Rochester, or a few other similarly favored communities.

Some medical men claim that the reason for this is that we are too commercialized; that dentists are conducting a business and not practicing a profession. That in our opinion is hardly fair, but at the same time the fact remains that dentists do little charity work of any kind.

There certainly must be a reason. Does the difference in age explain it? It is because medicine is an ancient profession and has built up a tradition of sacrifice and community service, while dentistry being only about seventy-five years of age may still possess the common failings of youth—egotism and selfishness.—Editor Dental Facts.

This is an unkind, and, we think, an unnecessary comment. The dental profession as a whole, and in particular, has contributed relatively as much to benevolence as any other benevolent profession, save perhaps the clergy.

Not all physicians live up to the traditional healing code, any more than do the dentists all feel called to minister needed treatment to all who suffer. By way of an illustration, who ever heard of a dentist refusing to give relief to any one suffering with an aching tooth, because no fee was forthcoming, or for any similar illness that he could relieve? Unless the conditions were not imperative, or there were some real and impracticable conditions that could not be met, the dentist would act. These hypothetical cases are so essentially dissimilar that they can not be considered without taking into account their environments. As a matter of fact, dentists from time immemorial have reverenced the healing art, and kept in their foreground the same traditions and ideals that have been the fundamentals that demand the sacredness of relief for all human needs, when suffering could be prevented. The physician necessarily has more chance than the dentist to serve, because the physician's field is of a wider scope. But dentistry is a growing field, and when there is need to widen a discussion of such a question, we believe the dentists will co-operate to the limit. Traditions or ethical evasions will not cloud this issue, so why discuss it?

